# Interpreting Biomarker Discordance in Inflammatory Bowel Disease: Beyond Fecal Calprotectin and C-Reactive Protein

**DOI:** 10.3390/biomedicines14091883

**Published:** 2026-08-24

**Authors:** Lovre Martinovic, Roko Santic, Marko Kumric, Marino Vilovic, Dinko Martinovic, Josko Bozic

**Affiliations:** 1 Department of Pathophysiology, University of Split School of Medicine, Soltanska 2A, 21000 Split, Croatia; lovre.martinovic@mefst.hr (L.M.); roko.santic@mefst.hr (R.S.); marko.kumric@mefst.hr (M.K.); josko.bozic@mefst.hr (J.B.); 2 Laboratory for Cardiometabolic Research, University of Split School of Medicine, Soltanska 2A, 21000 Split, Croatia; 3 Department of Maxillofacial Surgery, University Hospital of Split, Spinciceva 1, 21000 Split, Croatia; dinko.martinovic@kbsplit.hr; 4 Department of Maxillofacial Surgery, University of Split School of Medicine, Soltanska 2A, 21000 Split, Croatia

**Keywords:** inflammatory bowel disease, biomarkers, fecal calprotectin, leucine-rich alpha-2 glycoprotein, intestinal barrier, fibrosis, therapeutic drug monitoring, discordance

## Abstract

Treat-to-target management in inflammatory bowel disease (IBD) combines symptoms, fecal and serum biomarkers, endoscopy, histology, and cross-sectional imaging, but these measures frequently diverge. Discordance may reflect analytical variation, timing, disease location, phenotype, comorbidity, or partially non-overlapping biological processes. We performed a critical narrative review using a structured PubMed/MEDLINE search, supplemented by citation chaining and publisher searches. Guidelines, systematic reviews, diagnostic studies, cohorts, randomized trials, and selected mechanistic studies were prioritized. Fecal calprotectin (FC) and lactoferrin primarily reflect intestinal neutrophilic inflammation, whereas C-reactive protein (CRP) and related serum indices reflect a nonlocalizing systemic response. The fecal immunochemical test (FIT) detects gastrointestinal bleeding, and leucine-rich alpha-2 glycoprotein (LRG) remains promising but insufficiently standardized. We distinguish five biological biomarker domains—namely, fecal–neutrophil; serum–systemic; epithelial/barrier; restitution/resolution; and fibrosis/extracellular matrix (ECM) remodeling—from symptoms and clinical indices, pharmacologic measurements, and phenotype-directed reference assessments. Circulating barrier, repair, and matrix-turnover markers remain investigational. Reactive therapeutic drug monitoring (TDM) for anti-tumor necrosis factor (anti-TNF) agents has the most mature evidence. Vedolizumab and ustekinumab show exposure–response associations, but actionable thresholds are unvalidated, and clinical TDM is not established for newer biologics or oral small molecules. After objective confirmation of disease activity, the framework may support phenotype-directed therapeutic decisions but is not a validated algorithm. Clinically important disagreement should prompt assessment of sampling, assay, timing, infection, medication-related confounding, and pretest probability before phenotype-directed endoscopy, histology, imaging, or reactive TDM is selected. A single discordant result should neither trigger treatment escalation nor exclude active or structural disease.

## 1. Introduction

Treat-to-target care in inflammatory bowel disease (IBD) relies on several measures that do not necessarily move in parallel. The second Selecting Therapeutic Targets in Inflammatory Bowel Disease consensus (STRIDE-II) places early symptomatic response and subsequent clinical remission alongside normalization of objective inflammatory biomarkers. Within this framework, normalization of C-reactive protein (CRP) and reducing fecal calprotectin (FC) to an acceptable range—approximately 100–250 µg/g depending on the intended outcome—serve as short-to-intermediate targets, whereas endoscopic healing remains a long-term target. Histological healing in ulcerative colitis (UC) and transmural healing in Crohn’s disease (CD) indicate greater depth of remission but are not formal treatment targets [1]. These thresholds are clinical guideposts rather than self-executing rules. The American Gastroenterological Association (AGA) guidance allows FC below 150 µg/g, normal lactoferrin, or normal CRP to help rule out active UC in patients who are in symptomatic remission. In CD, the reassurance provided by FC below 150 µg/g and CRP below 5 mg/L depends substantially on whether endoscopic remission has recently been documented. Conversely, either elevated biomarkers in an asymptomatic patient or persistent symptoms despite normal markers usually requires objective confirmation rather than an automatic change in therapy [2,3].

The expanding range of advanced therapies has intensified interest in moving beyond universal biomarker thresholds toward individualized treatment selection that integrates disease phenotype, objective inflammatory burden, prior treatment response, and potentially patient-specific inflammatory profiles. A recent exploratory conference study linked dominant inflammatory-marker patterns to differential biologic responses, but treatment was not assigned using marker results and the cohort comprised only 18 patients; these observations remain hypothesis-generating rather than a validated treatment-selection strategy [4].

How often discordance occurs, and how it should be interpreted, depends on phenotype, anatomical location, assay, cutoff, sampling time, and treatment. CRP may remain normal in active UC, particularly when disease is limited or does not provoke a marked systemic response [5,6]. FC generally performs better in colonic than in isolated small-bowel CD, but a low value cannot reliably exclude small-bowel inflammation. Dilution during luminal transit may contribute to this difference, although it has not been established as the sole explanation [7,8,9,10]. Normal noninvasive markers likewise reduce, but do not eliminate, the possibility of residual inflammatory or structural disease [11,12]. Once test quality, timing, and relevant confounders have been addressed, the direction of disagreement can nevertheless offer a useful clue about the process or anatomical site that should be examined next.

In this review, discordance denotes a clinically meaningful mismatch among symptoms, fecal or serum biomarkers, endoscopic or histological findings, cross-sectional imaging, and pharmacologic measurements. Existing treat-to-target and biomarker guidance defines preferred targets and when objective confirmation is required, but it does not offer a unified framework for interpreting why tests that sample different biological processes diverge. More specifically, STRIDE-II defines therapeutic targets and their timing, whereas AGA guidance defines when established biomarkers may suffice or require objective confirmation; the present framework addresses the intervening reasoning step after results disagree, without proposing new thresholds or monitoring schedules. We therefore separate biological biomarker domains from clinical overlays, drug-exposure measurements, and phenotype-directed reference assessments. The aim is not to create new phenotypes or thresholds, but to use the direction of discordance to formulate the next testable clinical question while preserving the hierarchy between validated and investigational measures [1,2,3,11].

## 2. Methodology

We conducted a critical narrative review, with reporting informed by the Scale for the Assessment of Narrative Review Articles (SANRA), to integrate clinical, translational, and mechanistic evidence across tests that differ in biological source, intended use, and validation stage [13]. A narrative design was selected because the review compares heterogeneous biomarkers, clinical measures, pharmacologic measurements, and reference assessments rather than estimating a single pooled intervention or diagnostic effect.

A structured core search was performed in PubMed/MEDLINE from database inception to 28 July 2026. The exact core query, using Medical Subject Headings (MeSH) and title/abstract fields, was (“Inflammatory Bowel Diseases”[MeSH Terms] OR “inflammatory bowel disease*”[Title/Abstract] OR “Crohn disease”[Title/Abstract] OR “Crohn’s disease” [Title/Abstract] OR “ulcerative colitis” [Title/Abstract]) AND (biomarker*[Title/Abstract] OR discordan*[Title/Abstract] OR “treat-to-target”[Title/Abstract] OR “mucosal healing” [Title/Abstract] OR “histologic healing”[Title/Abstract] OR “transmural healing” [Title/Abstract] OR “barrier function”[Title/Abstract] OR fibrosis [Title/Abstract] OR stricture*[Title/Abstract] OR “therapeutic drug monitoring”[Title/Abstract] OR “drug concentration*”[Title/Abstract] OR “anti-drug antibod*”[Title/Abstract] OR “loss of response”[Title/Abstract]). Marker-specific searches then paired the disease block with fecal calprotectin, fecal lactoferrin, C-reactive protein, fecal immunochemical test, leucine-rich alpha-2 glycoprotein, intestinal fatty acid-binding protein, citrulline, lipopolysaccharide-binding protein, soluble CD14, lipopolysaccharide, zonulin, regenerating islet-derived proteins 1A and 3A, interleukin-22, amphiregulin, trefoil factors, mucins, annexin A1, specialized pro-resolving mediators, matrix metalloproteinases, collagen neo-epitopes, YKL-40, galectin-3, vedolizumab, ustekinumab, interleukin-23p19 inhibitors, Janus kinase inhibitors, and sphingosine-1-phosphate receptor modulators. Backward and forward citation chaining from key guidelines, systematic reviews, and pivotal primary studies, together with targeted searches of PubMed and publisher records, was used to identify additional reports. Conference proceedings were considered only when they provided otherwise unavailable evidence, principally for emerging markers without a full publication. The broad PubMed search retrieved approximately 18,000 records and was used to map the literature rather than for exhaustive systematic screening. Stage-specific screening counts were not recorded.

Eligible sources were human IBD guidelines or consensus statements, systematic reviews and meta-analyses, randomized trials, prospective or retrospective cohorts, diagnostic-accuracy studies, and translational studies that compared a candidate measure with an objective disease assessment or clinically relevant outcome. Animal and in vitro studies were included only for mechanistic interpretation, and conference abstracts only when no full report was available. Candidate biomarkers were included when they represented a distinct biological domain relevant to a recurring discordance pattern and had at least one human IBD study permitting comparison with established inflammatory, endoscopic, histological, imaging, or outcome measures. Broad untargeted omics, microbiome classifiers, and artificial-intelligence-derived signatures were excluded unless they directly addressed a prespecified domain, because their model-specific outputs are not directly comparable with single-analyte discordance. Potentially relevant sources were assessed iteratively by title and abstract and, when relevant or uncertain, by full text; study design, population, assay, sampling timing, reference standard, outcome, proposed threshold, and major limitations were extracted. Within each domain, sources were retained when they directly compared a candidate measure with an objective disease assessment or clinically relevant outcome, clarified a mechanism central to a recurrent discordance pattern, or represented the highest-level or most recent human evidence. When multiple reports addressed the same point, guidelines, systematic reviews, prospective or diagnostic-accuracy studies, randomized trials, and externally validated findings were prioritized. No new meta-analysis, formal risk-of-bias assessment, or certainty assessment using the Grading of Recommendations Assessment, Development and Evaluation (GRADE) framework was undertaken; the evidence-maturity labels are descriptive, and selection bias and heterogeneity remain possible. Formal independent duplicate screening was not undertaken; accordingly, no prespecified inter-reviewer disagreement-resolution procedure was used. A Preferred Reporting Items for Systematic Reviews and Meta-Analyses (PRISMA) flow diagram was not prepared because the aim was critical narrative synthesis rather than exhaustive effect estimation. Quantitative estimates for leucine-rich alpha-2 glycoprotein were taken from the registered systematic review [14]. The final synthesis comprised 112 cited sources.

## 3. Biomarker Anchors and the Signals They Capture

Although FC, CRP, fecal bleeding markers, albumin, and platelet count are often grouped under “disease activity”, they capture different biological signals. Their sources, kinetics, anatomical sensitivity, and analytical limitations explain part of clinical discordance but do not account for all cases. Table 1 summarizes the principal readouts, limitations, and clinical readiness of established and emerging biomarkers. Clinical readiness is considered separately from biological domain, with routine clinical, emerging human-validated, and mechanistic or exploratory measures distinguished throughout.

### 3.1. Fecal Calprotectin and Lactoferrin

FC is the S100A8/S100A9 complex released predominantly by recruited neutrophils, although monocytes and macrophages also contribute. Stool concentrations therefore provide a close, although indirect, readout of intestinal neutrophilic inflammation rather than a direct quantitative measure of mucosal inflammatory flux [7,29]. A diagnostic-accuracy meta-analysis reported pooled sensitivity of approximately 85%, specificity of 75%, and an area under the curve (AUC) of 0.88 for endoscopic activity, with stronger performance in UC than in CD [30]. FC also varies substantially within the same patient, especially at higher concentrations and during active disease, while sampling time, storage, and assay choice add further variability. A borderline or surprising value may therefore be more informative when repeated under standardized conditions. FC is not disease-specific; enteric infection, nonsteroidal anti-inflammatory drug (NSAID) enteropathy, diverticular inflammation, neoplasia, and other gastrointestinal inflammatory conditions can all increase stool concentrations [7,18]. Its performance is lower and more heterogeneous in isolated small-bowel CD, particularly proximal disease, but FC is not blind to small-bowel inflammation, and a low result cannot exclude it when pretest probability remains high [8,9,10]. Fecal lactoferrin reflects a similar neutrophil-dominant process and has broadly comparable diagnostic performance in some settings, although it is less standardized, less widely available, and supported by more limited evidence in isolated small-bowel disease [15,16,17].

### 3.2. C-Reactive Protein and Erythrocyte Sedimentation Rate

CRP provides a more systemic view of inflammation. It is produced predominantly by hepatocytes within an interleukin-6 (IL-6)-dominant, cytokine-modulated acute-phase response and has a plasma half-life of approximately 19 h. Unlike FC, it does not localize inflammation to the intestine or quantify intestinal cytokine spillover [5,6]. CRP is elevated more often in active CD than in UC and may remain normal despite endoscopically active disease. Host and genetic factors can attenuate the response, although no clinically applicable prevalence has been established for a genetically defined “low-producer” subgroup, and routine genotyping is not recommended. In one cohort of extensive UC, CRP correlated more closely than the fecal markers studied with colon-wide inflammatory burden, but that finding should not be extrapolated to all UC phenotypes [31,32]. The erythrocyte sedimentation rate (ESR) captures a broader and slower-moving signal generated by fibrinogen, immunoglobulins, and red-cell behavior, and its interpretation is readily influenced by age, sex, anemia, and hypoalbuminemia [6,18].

### 3.3. Albumin and Platelets

Albumin and platelet count are most useful when treated as contextual measures rather than direct surrogates of mucosal activity. A low albumin concentration may reflect inflammation-related changes in synthesis, distribution, and catabolism, together with capillary or enteric protein loss. Malnutrition may coexist, but albumin is neither a direct nutritional marker nor a rapidly responsive activity measure; its long half-life makes it more indicative of subacute disease burden [19]. Reactive thrombocytosis may accompany inflammation through IL-6 and thrombopoietin signaling, although iron deficiency is an important independent driver and often complicates interpretation [20,21]. Both measures add information about systemic, protein, and hematologic physiology, but neither should be read as an isolated measure of intestinal inflammation [19,20,21,33].

### 3.4. Leucine-Rich Alpha-2 Glycoprotein

Leucine-rich alpha-2 glycoprotein (LRG) is among the more clinically developed emerging serum inflammatory markers, particularly in Japan. It is induced through several inflammatory pathways and can originate from hepatocytes, neutrophils, and inflamed intestinal tissue, although the proportional contribution of each source to circulating LRG is uncertain [22,34,35]. Multiple cohorts have reported associations with clinical or endoscopic activity, including in some patients with normal CRP and during adalimumab treatment [23,24,36,37,38,39,40]. The 2025 systematic review and meta-analysis included 14 studies and 1794 patients, all from Japan. It supported moderate diagnostic performance but did not establish pooled head-to-head superiority over CRP in both UC and CD [13]. A retrospective small-bowel CD study reported sensitivity of 72.1% and specificity of 93.7% at an assay-specific cutoff of 16.3 µg/mL, a finding that requires independent external validation [41]. Evidence remains geographically concentrated, assays and thresholds are not internationally standardized, and no randomized trial has shown that LRG-guided treatment improves outcomes. Stenosis has been associated with LRG in one cohort, but available data do not establish fibrosis specificity or elevation from remodeling in the absence of inflammation [23,25].

### 3.5. Fecal Immunochemical Test

The fecal immunochemical test (FIT) detects human globin and therefore gastrointestinal bleeding rather than a UC-specific biological compartment. In UC, FIT correlates with endoscopic activity, can help predict relapse, and may complement FC for endoscopic and histological assessment [26,27,28]. FIT remains nonspecific for the source of bleeding; a negative result does not exclude nonbleeding inflammation, and its principal evidence base is colonic disease. In quiescent UC, FC has outperformed FIT for predicting mucosal healing in a prospective multicenter study [42].

### 3.6. Pre-Interpretive Assessment of Discordance

Before biological meaning is assigned to discordance, the credibility of the mismatch should be assessed. Relevant considerations include timing relative to symptoms, treatment, bowel preparation, surgery, and intercurrent illness; specimen handling and storage; assay platform and cutoff; and alternative explanations such as Clostridioides difficile infection, other enteric infections, nonsteroidal anti-inflammatory drug (NSAID) exposure, gastrointestinal bleeding, and non-IBD inflammation. Repetition of an unexpected or borderline FC measurement under standardized conditions is appropriate when clinically safe. Conversely, in selected patients with stable symptomatic remission and a documented phenotype, concordantly reassuring FC and CRP or lactoferrin results may reduce the need for routine endoscopic reassessment; this is a risk-based triage strategy, not a substitute for endoscopy when markers are discordant, persistently abnormal, or potentially insensitive to the suspected anatomical location. The negative predictive value of normal markers is lower in small-bowel, postoperative, perianal, and structurally complicated disease, particularly when objective remission has not been documented [2,3,11,18].

## 4. How Discordance Presents in Practice

Patterns A–E are unvalidated heuristic constellations intended to structure a differential interpretation, not to define phenotypes, diagnose a mechanism, or prescribe management. Patterns A–C arise from symptoms, FC, and CRP; D and E require histology, specialized barrier assessment, or structural imaging. Each remains probabilistic, phenotype-dependent, and contingent on pretest probability and prior investigations. Table 2 therefore presents illustrative, context-dependent assessments rather than standard clinical responses.

### 4.1. Pattern A: High Symptoms with Low FC and Low CRP

Low inflammatory markers make active colonic neutrophilic inflammation or a marked systemic response less likely, but do not establish inactive IBD. Functional overlap, bile-acid diarrhea, dysmotility, small-intestinal bacterial overgrowth, infection, postoperative anatomy, and structural disease remain alternatives [11,49]. Low FC is also less reassuring in isolated small-bowel disease [3,7,10]. Persistent or red-flag symptoms warrant phenotype-directed endoscopy or imaging rather than empiric immunosuppression.

### 4.2. Pattern B: Low or Absent Symptoms with High FC

Persistent elevation may indicate subclinical neutrophilic activity and is associated with subsequent relapse [43]. Infection, NSAID exposure, and sampling variation should be addressed, and an unexpected result repeated when appropriate. Endoscopy or targeted imaging should confirm sustained elevation before treatment is changed [2,3].

### 4.3. Pattern C: Fecal–Systemic Mismatch

The direction matters. High CRP with low FC (C1) suggests a systemic or extraintestinal source, infection, abscess or penetrating disease, or small-bowel activity, although colonic inflammation remains possible [5,7,50]. High FC with low CRP (C2) is compatible with predominantly mucosal inflammation and a limited systemic acute-phase response. In either direction, the source should be clarified with phenotype-appropriate assessment; LRG may add context but cannot localize disease.

### 4.4. Pattern D: Discordance Among Endoscopy, Histology, and Barrier Function

Histological activity may persist beneath an endoscopically normal mucosa and predicts relapse in UC [51]. Separately, barrier dysfunction defined by confocal laser endomicroscopy (CLE) may persist despite conventional endoscopic and histological remission and was associated with adverse outcomes in the ERIca cohort [44]. A later study from the same setting suggested prognostic value beyond transmural healing [45]. These findings support biological non-equivalence rather than routine CLE-guided treatment; CLE remains specialized, and circulating barrier markers cannot replace biopsy.

### 4.5. Pattern E: Low Inflammatory Markers with Structural or Transmural Disease

Low FC and CRP are least reassuring in stricturing, penetrating, perianal, or anatomically restricted disease, which may also be missed by mucosal endoscopy. Collagen-turnover studies support phenotype association and risk prediction, but these are not validated for monitoring [46,47,48]. Intestinal ultrasound (IUS) and magnetic resonance enterography (MRE) remain the clinical reference assessments, whereas circulating extracellular matrix (ECM) assays remain investigational.

## 5. Separating Biological Biomarker Domains from Interpretive Layers

**Figure 1:**
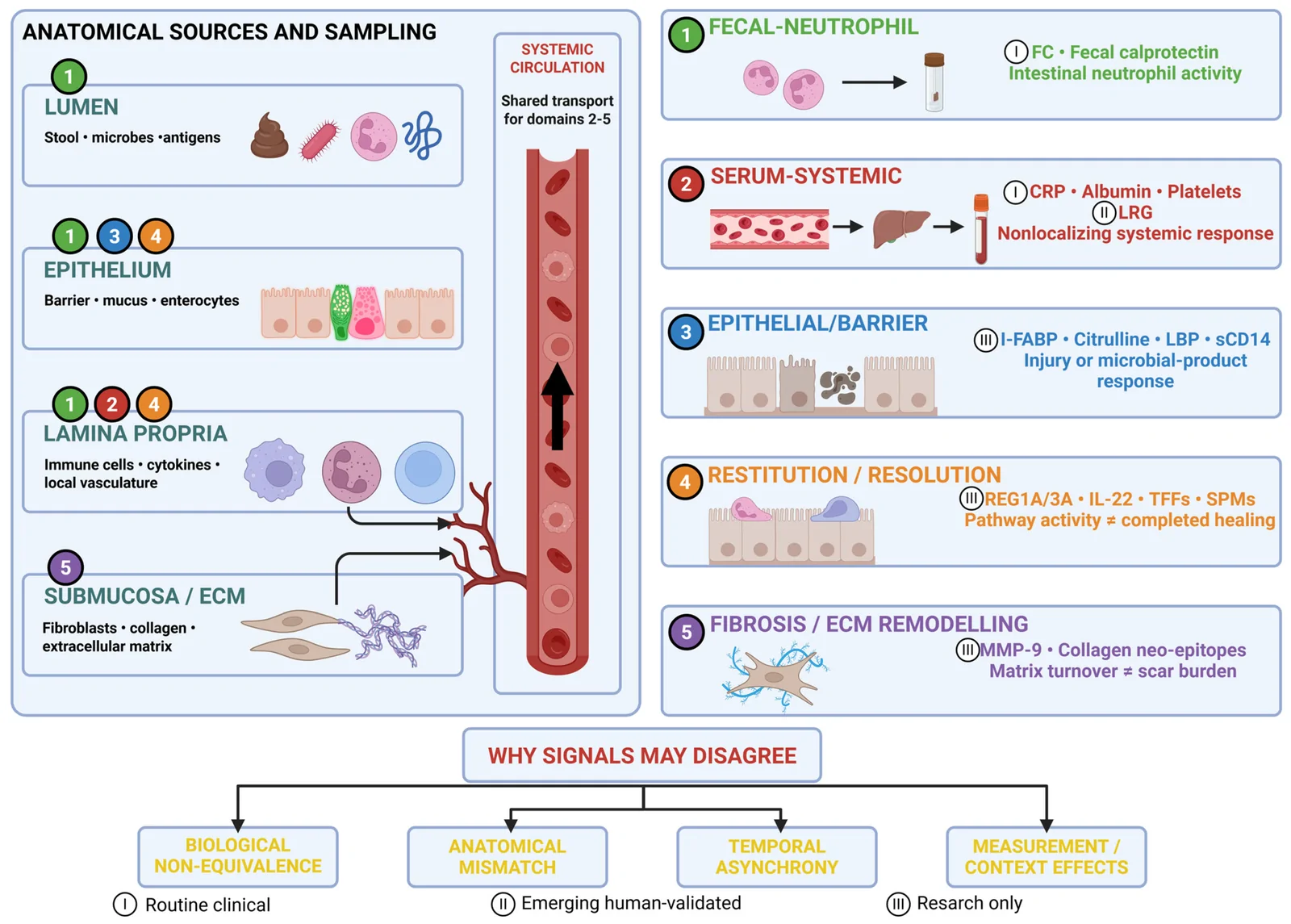
Anatomical sources, overlapping functional biomarker domains, evidence-maturity tiers, and major mechanisms underlying biomarker discordance in inflammatory bowel disease. The left panel depicts the lumen, epithelium, lamina propria, submucosa/extracellular matrix, and systemic circulation. Numbered circles identify the predominant anatomical sources or readouts associated with the five functional domains; these assignments are not mutually exclusive. The right panel summarizes (1) the fecal–neutrophil domain, represented by fecal calprotectin and intestinal neutrophil activity; (2) the serum–systemic domain, comprising routine nonlocalizing systemic measures and the emerging marker LRG; (3) the epithelial/barrier domain, represented by candidate markers of enterocyte injury and microbial-product exposure or host response; (4) the restitution/resolution domain, comprising candidate pathway-activity markers that do not establish completed healing; and (5) the fibrosis/ECM-remodeling domain, comprising markers of matrix turnover that do not quantify fixed scar burden. LBP and sCD14 also overlap functionally with the serum–systemic domain, whereas MMP-9 may reflect both neutrophilic inflammation and ECM turnover. Roman numerals indicate clinical readiness: I, routine clinical measures; II, emerging human-validated measures with incomplete standardization or limited availability; and III, research-only candidates. These tiers are descriptive and do not constitute formal certainty grading. The lower panel summarizes four major sources of discordance: biological non-equivalence, anatomical mismatch, temporal asynchrony, and measurement or context effects. The framework is conceptual and is not a validated diagnostic classification or treatment algorithm. Abbreviations: CRP, C-reactive protein; ECM, extracellular matrix; FC, fecal calprotectin; I-FABP, intestinal fatty acid-binding protein; IL-22, interleukin-22; LBP, lipopolysaccharide-binding protein; LRG, leucine-rich alpha-2 glycoprotein; MMP-9, matrix metalloproteinase-9; REG, regenerating islet-derived protein; sCD14, soluble CD14; SPMs, specialized pro-resolving mediators; TFFs, trefoil factors. Created in BioRender. Kumric, M. (2026) https://BioRender.com/f2ed8zr.

To avoid treating non-equivalent tests as interchangeable, the framework separates five biological biomarker domains from clinical overlays, pharmacologic measurements, and reference or functional assessments. The domains are fecal–neutrophil, serum–systemic, epithelial/barrier, restitution/resolution, and fibrosis/ECM-remodeling. Symptoms and clinical indices form a clinical overlay; drug concentrations and anti-drug antibodies (ADAs) are pharmacologic measurements; and endoscopy, histology, cross-sectional imaging, CLE, and permeability testing are reference or functional assessments rather than biomarkers. Table 3 formalizes these distinctions, whereas Figure 1 traces the measurement pathway from underlying biology to the observed clinical signal and identifies four major sources of disagreement: biological non-equivalence, anatomical mismatch, temporal asynchrony, and measurement or context effects. The domain boundaries and proposed mechanisms remain conceptual and require prospective validation. These domains are functional interpretive categories rather than biologically discrete or mutually exclusive compartments. Assignment indicates the predominant readout relevant to a discordance question, not an exclusive source or mechanism. Cross-domain behavior is expected: MMP-9 participates in neutrophilic inflammation and ECM turnover, whereas LBP and sCD14 reflect microbial-product exposure together with a systemic host response. Figure 1 maps the five biological biomarker domains onto their principal anatomical compartments and sampling routes and summarizes how biological non-equivalence, anatomical mismatch, temporal asynchrony, and measurement or context effects can generate discordant results, whereas Table 3 distinguishes these domains from clinical overlays, pharmacologic measurements, and reference or functional assessments.

Clinical readiness is distinct from biological domain. We distinguish three pragmatic tiers: (i) established clinical measures, comprising FC and CRP together with context-dependent use of fecal lactoferrin, FIT, ESR, albumin, and platelet count; (ii) emerging measures with human validation but incomplete standardization or geographically limited availability, principally LRG; and (iii) mechanistic or exploratory candidates, comprising circulating epithelial/barrier, restitution/resolution, and ECM-remodeling analytes. Only the first tier currently has routine roles supported by clinical guidance. Emerging and exploratory measures lack validated treatment thresholds and evidence that measurement-guided intervention improves outcomes.

FC and lactoferrin anchor the fecal–neutrophil domain and primarily reflect intestinal neutrophil recruitment and degranulation. Their performance varies with disease location and non-IBD intestinal inflammation, and neither measures fibrosis, barrier competence, or drug exposure [7,15,30].

CRP, ESR, albumin, platelet count, and LRG occupy the serum–systemic domain. They capture different components of acute-phase signaling, protein homeostasis, and hematologic physiology, but do not localize the source of inflammation and may change with infection, extraintestinal disease, or comorbidity [5,19,20,22].

The epithelial/barrier biomarker domain includes intestinal fatty acid-binding protein (I-FABP), citrulline, lipopolysaccharide-binding protein (LBP), soluble CD14 (sCD14), and lipopolysaccharide (LPS). These analytes represent enterocyte injury, functional enterocyte mass, or systemic exposure and response to microbial products; they are not interchangeable permeability tests and have no validated treatment thresholds [52,53,54,63,64]. CLE and multisugar permeability tests belong to the separate category of specialized functional assessments. LBP and sCD14 therefore overlap functionally with the serum–systemic domain.

Regenerating islet-derived (REG) proteins, interleukin-22 (IL-22), amphiregulin, trefoil factors, mucins, annexin A1, and specialized pro-resolving mediators (SPMs) populate the restitution/resolution domain. Because their concentrations may rise during injury, compensation, regeneration, or ongoing inflammation, no single direction of change can presently be interpreted as completed healing [55,65,66,67].

Circulating matrix metalloproteinases (MMPs), collagen neo-epitopes, YKL-40 (chitinase-3-like protein 1), and galectin-3 form an investigational fibrosis/ECM-remodeling domain and may reflect matrix turnover or inflammation [46,47,57,68,69,70]. Structural disease itself is assessed clinically by IUS, MRE, computed tomography enterography, endoscopy, or surgical evaluation. This label denotes a predominant use in studies of ECM turnover and structural phenotypes, not validated specificity for fixed intestinal fibrosis or accumulated scar burden.

Symptoms and clinical indices remain essential measures of patient burden but can diverge from objective inflammation because of functional disorders, bile-acid diarrhea, dysmotility, infection, postoperative anatomy, or fixed structural complications. Drug concentrations and ADAs similarly describe exposure and immunogenicity rather than disease location; their interpretation is useful only within a defined treatment question [58].

Accordingly, discordance should first be interpreted by determining whether the two tests sample the same process. When they do not, neither result should be privileged by default. A phenotype-directed reference assessment should instead be selected to adjudicate the unresolved biological or anatomical question.

## 6. Investigational Epithelial/Barrier Markers Beyond Conventional Inflammatory Activity

Inflammation and barrier dysfunction often coexist but are not equivalent states of recovery. Endoscopy, histology, permeability testing, and transmural imaging interrogate different layers, so normalization of one does not ensure normalization of the others [61,62]. In the ERIca study, CLE-defined barrier healing was associated with fewer subsequent major adverse outcomes and added prognostic information beyond endoscopic and histological remission [44]. A later, smaller study from the same setting suggested prognostic value beyond transmural healing [45]. These findings support barrier function as a research endpoint, but not a universal treatment target; CLE remains invasive, operator- and protocol-dependent, and insufficiently validated across centers.

The candidate blood markers discussed in this context capture different aspects of barrier biology rather than a common permeability signal [52]. I-FABP, for example, is a cytosolic protein released after enterocyte membrane injury and is expressed predominantly by mature small-intestinal enterocytes. Concentrations are increased in pediatric CD, but the weak correlation with FC seen in one cohort shows limited agreement in that dataset; it does not prove that the two analytes represent wholly independent processes [52]. Interpretation is further complicated by food intake, diurnal variation, renal handling, and the amount of viable epithelium available to release the protein. With advanced epithelial loss, the relation between injury and circulating I-FABP may therefore become nonlinear or even yield a low signal despite severe disease [52]. For now, I-FABP is best considered an exploratory marker of enterocyte injury, not a validated test of small-bowel activity or healing.

Plasma citrulline reflects functional enterocyte mass because it is produced largely by small-bowel enterocytes. Its most established clinical context is extensive loss of absorptive surface rather than routine IBD monitoring [63,64]. Renal function, nutritional state, and inflammation-related suppression of enterocyte metabolism influence its concentration, and no validated IBD threshold distinguishes active inflammation, fixed structural loss, and postoperative reduction in bowel length. In particular, a low citrulline concentration with normal CRP or minimally elevated FC should not be interpreted as a molecular signature of quiescent fibrostenotic CD without phenotype-appropriate imaging.

LBP, sCD14, and circulating LPS sit one step further downstream: they reflect exposure or host response to microbial products, not permeability itself. LBP is a hepatic acute-phase protein, whereas sCD14 is released mainly by monocytes and macrophages, and both may rise in systemic inflammation, infection, and other non-IBD conditions. Associations with CD activity, including persistence in some clinically inactive patients, are compatible with ongoing microbial exposure but neither identify the intestinal source nor prove a persistent epithelial defect [54,71,72]. The reported links between circulating LPS, disease severity, and acute-phase activation are biologically plausible [73,74,75]. However, direct LPS measurement is particularly susceptible to contamination, matrix effects, and preanalytical variation. It is therefore more accurate to describe these analytes as candidate markers of microbial translocation or the host response to it than as direct permeability tests.

Serum “zonulin” requires particular caution. Widely used commercial enzyme-linked immunosorbent assays do not reliably quantify pre-haptoglobin-2 and may instead detect properdin or related proteins; their results cannot be assumed to represent either zonulin concentration or intestinal permeability [76,77,78]. Tissue claudins and occludin are mechanistically informative but usually require biopsy, while proposed circulating tight-junction fragments remain insufficiently validated [62,79,80]. Functional multisugar permeability testing and CLE are closer to direct barrier assessment, but neither is a routine IBD treatment target. No circulating candidate in this section has standardized assays, externally validated decision thresholds, or evidence that marker-guided treatment improves outcomes; these measures remain research adjuncts.

## 7. Mechanistic and Exploratory Signals of Epithelial Repair and Inflammatory Resolution

Epithelial repair and inflammatory resolution overlap but do not necessarily proceed in parallel. Repair restores the epithelial and mucus barriers, whereas resolution requires leukocyte recruitment to subside, inflammatory cells and debris to be cleared, and tissue homeostasis to return. Both processes may continue after CRP and FC have fallen, yet the timing and direction of candidate circulating signals remain too poorly defined to infer successful repair from a single measurement [55,65,81]. Reviews have likewise not identified a circulating analyte that can serve as a stand-alone marker of complete mucosal healing [66,82]. The molecules discussed below are therefore better viewed as candidate markers or biological effectors than as evidence for a clinically established “repair compartment”.

IL-22 is a useful example of why pathway activation cannot be equated with healing. Signaling through the epithelial IL-22 receptor can promote proliferation, antimicrobial-peptide expression, and mucus production, but the net effect varies with inflammatory context, receptor availability, and endogenous IL-22 binding protein. Small observational studies have linked serum IL-22 to UC activity, without validating it as a substitute for endoscopy or a direct measure of repair [83]. In a phase Ib study, the interleukin-22 fusion-protein agonist efmarodocokin alfa showed epithelial target engagement and increased REG3A, together with preliminary safety and pharmacokinetic data [84]. The subsequent randomized phase II trial, however, found no statistically significant advantage over placebo for clinical remission, clinical response, endoscopic healing, or endoscopic remission despite pharmacodynamic pathway activation [56]. Thus, an increase in IL-22-responsive proteins, including REG3A, does not by itself demonstrate restoration of mucosal integrity.

Amphiregulin is an epidermal growth factor receptor ligand produced by several immune and stromal populations. Experimental data support epithelial protection and regulatory T-cell function [85,86]. However, Th17-derived amphiregulin can also activate intestinal myofibroblasts and promote fibrosis [87]. Its concentration or tissue expression is therefore context-dependent and not a unidirectional healing signal. Trefoil factors similarly facilitate restitution in experimental systems, yet circulating trefoil factor 3 (TFF3) failed to identify mucosal healing in patients with CD receiving tumor necrosis factor antagonists [88]. Mucins and goblet-cell programs are altered in UC, and region-specific tissue mucin signatures may aid biological stratification [89,90,91]. However, these are tissue-based findings rather than validated serum or fecal repair assays.

REG proteins are among the better-studied candidates at the interface of epithelial stress and regeneration. Even so, available studies mainly link them to inflammation, target engagement, or subsequent risk, not to completed epithelial repair. Serum REG1A correlated with endoscopic activity, particularly in UC, and was proposed as an activity biomarker [92]. A conference report in CD associated higher REG3A with adverse disease progression, but the derived threshold had only modest discrimination and requires full publication and external validation [93]. REG gene expression is increased in inflamed mucosa [67,94]. A high circulating concentration may therefore represent ongoing epithelial injury with a compensatory regenerative response rather than effective healing. Conversely, a falling value cannot yet be assumed to indicate failure of repair. Longitudinal studies with prespecified sampling, histological assessment, and durable clinical outcomes are needed before direction-of-change rules can be proposed.

Annexin A1 and specialized pro-resolving mediators (SPMs), including resolvins, protectins, and maresins, participate in terminating inflammation and supporting tissue recovery [65,81,95]. SPMs occur at low concentrations and are chemically unstable; validated liquid chromatography–tandem mass spectrometry is preferable to immunoassays prone to cross-reactivity. Across this domain, no candidate has validated clinical cutoffs or evidence that measurement-guided treatment improves outcomes. Their present role is mechanistic or translational stratification, not declaration of healing or selection of escalation or de-escalation.

## 8. Extracellular Matrix Remodeling and Transmural Structural Disease

Fibrostenosing CD rarely separates neatly into inflammation or fibrosis; most lesions contain both in varying proportions. ECM turnover is a dynamic process and is not equivalent to the amount of established scar within a stricture. ECM deposition may persist after acute inflammatory activity subsides, yet low CRP and FC do not prove that a stricture is mainly fibrotic because both biomarkers may miss anatomically restricted or proximal small-bowel disease. Conversely, elevated inflammatory markers say little about how much fibrosis is present within a stenosis. Clinically, the more useful questions are whether luminal narrowing, bowel-wall thickening, prestenotic dilatation, impaired passage, or penetrating disease is present and how much active inflammation accompanies the structural lesion.

In practice, cross-sectional imaging remains the cornerstone of structural assessment. IUS and MRE are preferred for serial evaluation where expertise and access permit; computed tomography enterography is useful when rapid or more widely available imaging is needed, with due attention to radiation exposure. A useful report describes stricture location and length, wall thickness, luminal narrowing, prestenotic dilatation, vascularity, and any penetrating complication. Imaging can track structural progression and provide an estimate of inflammatory activity, but it cannot yet quantify fibrosis accurately enough to replace multidisciplinary judgment. Elastography and advanced magnetic-resonance techniques are promising, although standardization is incomplete [60]. Assessment should therefore remain grounded in targeted imaging, endoscopy when the segment is traversable and the procedure safe, and early surgical input when obstruction or complications are suspected.

Serum ECM assays remain far less mature than imaging. Matrix metalloproteinases and their inhibitors participate in matrix degradation and remodeling [57]. However, serum MMP-9 is also strongly linked to neutrophilic and inflammatory activity. Its reported association with relapse in quiescent CD without correlation with CRP does not establish it as a fibrosis-specific marker [68]. MMP-9 is better understood as a cross-domain inflammatory and ECM-turnover candidate than as a direct measure of scar burden. YKL-40 and galectin-3 likewise reflect inflammation and tissue remodeling across multiple organs and diseases; neither has demonstrated sufficient specificity or validated thresholds for IBD fibrosis [69,70]. Tissue transforming growth factor beta (TGF-β) signaling is central to fibrogenesis but remains a mechanistic pathway rather than an established noninvasive biomarker.

Neo-epitope assays of collagen formation and degradation offer a more targeted approach to matrix turnover. These include type III and IV collagen formation markers (PRO-C3 and PRO-C4) and matrix metalloproteinase-generated type III and IV collagen degradation markers (C3M and C4M). Associations with stricturing or penetrating phenotype and subsequent progression have been reported [46,96]. However, these signals may still reflect systemic matrix turnover and inflammation, and assay-specific thresholds have not been externally validated. In a pre- and post-resection study, a model combining type III, V, and VI collagen formation markers (PRO-C3, PRO-C5, and PRO-C6) discriminated stenotic from luminal CD with an AUC of approximately 0.91, while PRO-C6 showed the clearest association with submucosal fibrosis [47]. This was a derivation result rather than independent clinical validation and should not be generalized to a PRO-C3/C4M panel. Plasma collagen type III alpha-1 chain was associated with later strictures in a pediatric cohort [48]. However, it is not analytically equivalent to the PRO-C3 formation neo-epitope.

No serum ECM panel can currently diagnose a fibrotic stricture, reliably separate reversible inflammation from fixed fibrosis, or replace IUS, MRE, endoscopy, and surgical assessment. Such assays should not determine biologic escalation, dilation, or surgery; their appropriate role is prospective phenotyping and antifibrotic trials with centralized imaging, histopathology when available, prespecified cutoffs, and external validation.

## 9. Integrating Drug Exposure with Biomarker Discordance

### 9.1. Scope and Limitations of Therapeutic Drug Monitoring

Within this framework, pharmacologic measurements answer a different question from inflammatory biomarkers and reference assessments. FC, CRP, endoscopy, and imaging establish whether and where activity persists; drug concentrations and anti-drug antibodies (ADAs) may then help explain loss of response in a treated patient. A low drug concentration or detectable ADA does not establish active IBD and becomes clinically actionable only after activity has been objectively confirmed and infection, noninflammatory symptoms, nonadherence, and structural complications have been considered. In that setting, reactive drug-concentration and ADA testing is most clinically useful for anti-tumor necrosis factor (anti-TNF) therapy, where it can distinguish probable pharmacokinetic from mechanistic failure [58]. Sampling should occur at an interpretable time point, preferably at trough when a trough target is used, and serial results should rely on the same or a demonstrably comparable assay.

Randomized evidence does not support universal concentration-guided anti-TNF maintenance. The Trough Concentration Adapted Infliximab Treatment (TAXIT) trial and the Tailored Treatment With Infliximab for Active Crohn’s Disease (TAILORIX) trial did not demonstrate superiority for their primary remission endpoints [97,98]. Observational data remain supportive but susceptible to selection and management bias [99]. A meta-analysis of randomized trials found no clear benefit of routine proactive TDM for maintaining remission [100]. The Norwegian Drug Monitoring (NOR-DRUM) trials were negative during induction and positive during maintenance across several immune-mediated diseases, which limits direct extrapolation to IBD [101,102]. The 2024 European Crohn’s and Colitis Organisation (ECCO) guideline for CD therefore found insufficient evidence to recommend proactive anti-TNF TDM over reactive monitoring or standard care, while allowing TDM as an aid to dose optimization [59].

Drug concentration and ADA status are most informative when interpreted together and against a drug-, assay-, phenotype-, and outcome-specific range. Low concentration without ADA suggests nonimmune pharmacokinetic failure and should prompt review of adherence, inflammatory burden, albumin, protein loss, body size, and clearance. Low concentration with low-titer ADA may still respond to dose or interval optimization and, in selected anti-TNF-treated patients, immunomodulator optimization. Low or undetectable concentration with persistent high-titer ADA favors immune-mediated pharmacokinetic failure and usually a switch, whereas objective inflammation despite an adequately interpreted concentration suggests probable mechanistic failure and favors a change in mechanism [58,97,98].

Adalimumab concentrations measured at secondary loss of response did not reliably predict response to subsequent intensification in a multicenter retrospective study, illustrating that TDM informs probability rather than determining treatment [103]. The human leukocyte antigen (HLA)-DQA1*05 allele may increase anti-TNF immunogenicity risk in some populations, but findings are inconsistent, and genotyping is not a substitute for drug and ADA measurement [104,105].

Evidence maturity differs substantially across other therapeutic classes. Vedolizumab has an exposure–response association, particularly in UC, but proposed induction and maintenance concentrations overlap and have not been validated as treatment thresholds in randomized level-guided trials [106]. For ustekinumab, a meta-analysis of observational studies found higher trough concentrations among clinical remitters, whereas endoscopic evidence was limited and prospective benefit from concentration-guided adjustment remains unproven [107]. International expert reviews therefore regard non-anti-TNF TDM as an important research priority rather than an established routine strategy [108]. For interleukin-23p19 inhibitors, Janus kinase inhibitors, and sphingosine-1-phosphate receptor modulators, exposure–response information is derived mainly from development programs; standardized commercial assays and clinically actionable thresholds are generally unavailable [109].

### 9.2. Biosimilars in Pharmacologic Interpretation

Biosimilars now constitute an important component of biologic treatment in IBD, and available evidence generally supports efficacy, safety, and immunogenicity comparable to their reference biologics while improving affordability and access [110]. Their use does not create a separate biomarker domain: when loss of response is suspected, the same sequence of objective disease confirmation followed by context-specific reactive TDM applies. Product identity, switch history, sampling time, and assay continuity should be documented, and symptoms, inflammatory biomarkers, drug concentration, ADAs, and disease phenotype should be interpreted together rather than attributing loss of response to the switch alone. Biosimilar-specific therapeutic thresholds have not been validated.

### 9.3. Clinical Adjudication of Discordant Results

The proposed sequence begins with verification of the mismatch as described in Section 3.6, followed by interpretation of symptoms, FC, and CRP as a pattern and selection of the phenotype-directed assessment that can adjudicate the unresolved question. Ileocolonoscopy with biopsies addresses colonic or ileocolonic mucosal and microscopic disease; IUS or MRE addresses small-bowel, transmural, or stricturing disease; and pelvic magnetic resonance imaging (MRI) addresses perianal disease. The phenotype-specific applications of this sequence are summarized in Table 2.

Pharmacologic measurements therefore enter only after a phenotype-matched objective assessment has confirmed ongoing activity and only when the result would alter a specific treatment decision. In this sequence, inflammatory biomarkers, endoscopy, or imaging establish the inflammatory or structural target, whereas drug concentration and ADA results help classify probable nonimmune pharmacokinetic, immune-mediated pharmacokinetic, or mechanistic failure. This interpretation is most mature for anti-TNF therapy and remains less standardized for other therapeutic classes [58,106,107,108,109].

Barrier, restitution/resolution, and circulating ECM analytes remain research adjuncts. Therapeutic decisions should integrate objectively confirmed inflammatory activity, endoscopic or imaging findings when indicated, drug concentrations, ADA status, disease phenotype and behavior, prior response, safety, and patient preference. TDM is one pharmacologic layer rather than an autonomous decision rule, and the framework itself requires prospective validation.

## 10. Discussion

The principal contribution of this review is not another biomarker hierarchy, but a distinction among biological biomarker domains, clinical overlays, pharmacologic measurements, and reference assessments. Existing treat-to-target guidance appropriately defines targets and when objective confirmation is needed; the present framework addresses the intervening reasoning problem of why tests diverge and which assessment can resolve the mismatch. This distinction prevents a normal CRP, elevated FC, low drug concentration, or abnormal imaging result from being treated as interchangeable evidence [1,2,3,11]. In short, current guidance primarily answers what to monitor and when to confirm, whereas this framework addresses why measures may disagree and how that disagreement should determine the next phenotype-matched question.

The framework also makes evidence maturity explicit. Established fecal and serum markers can support monitoring but remain location- and context-dependent, whereas LRG is constrained by geographic concentration, assay heterogeneity, and absent outcome-guided trials [14,24,25]. Barrier and repair candidates may capture biology not represented by FC or CRP, yet target engagement is not equivalent to clinically meaningful healing, as shown by the IL-22/REG3A development program [56,84]. Circulating ECM-remodeling markers likewise provide turnover or phenotype signals without quantifying fixed scars or replacing imaging or histology.

Several limitations remain. The narrative design permits integration across heterogeneous evidence but cannot eliminate selection bias, and no formal risk-of-bias or certainty grading was performed. The proposed domains overlap biologically, candidate analytes may arise from multiple tissues, patterns A–E have not been prospectively validated, and evidence is uneven across disease phenotypes and therapeutic classes. The framework should therefore be treated as a testable conceptual model, not a diagnostic classification or treatment algorithm.

### Multimodal Biomarker Integration and Artificial Intelligence

Future studies should prospectively define discordance patterns, standardize sampling and assays, use phenotype-matched reference assessments, report incremental value beyond FC and CRP, and evaluate whether marker-informed decisions improve outcomes. Prospective trials should embed validated multimodal profiles within prespecified therapeutic-selection algorithms and compare algorithm-guided care with standard treat-to-target management using clinical, endoscopic, structural, safety, and resource-use outcomes. For drug exposure, trials should separate anti-TNF from non-anti-TNF agents and distinguish association with response from benefit of level-guided intervention.

Artificial intelligence could support this agenda by integrating longitudinal symptoms, FC and CRP trajectories, endoscopic and histological activity, imaging features, drug concentrations, ADAs, disease phenotype, and treatment history to estimate the probability and source of discordance and prioritize confirmatory assessment. Multi-omics machine learning has demonstrated technical feasibility for diagnostic classification and molecular subgrouping, but not improved therapeutic selection or safe replacement of endoscopy [111,112]. Clinical values will require harmonized inputs, transparent handling of missing and time-dependent data, external and prospective validation, calibration, interpretability, subgroup fairness, and decision-impact trials. Until then, such models should remain research decision support rather than autonomous treatment selectors or substitutes for phenotype-directed reference assessment.

## 11. Conclusions

Biomarker discordance in IBD is common because symptoms, fecal and serum biomarkers, pharmacologic measurements, and endoscopic, histological, or imaging assessments sample different processes. Clinically important disagreement should first prompt verification of timing, specimen quality, assay comparability, infection, medication exposure, comorbidity, and disease phenotype, followed by the reference assessment best matched to the unresolved question.

FC and CRP remain the principal noninvasive anchors; LRG and other emerging biomarkers may add context but cannot yet direct treatment. Reactive anti-TNF TDM has the most mature clinical role, whereas actionable thresholds remain unvalidated for vedolizumab, ustekinumab, interleukin-23p19 inhibitors, and oral small molecules. The multi-domain framework is intended to structure hypothesis generation, not to replace clinical judgment; no single discordant result should independently trigger escalation or exclude active or structural disease.

## Figures and Tables

**Table 1:** Established and emerging IBD biomarkers: biological readout, limitations, and clinical readiness.

Biomarker	Predominant Source/Readout	What It Can Indicate	Principal Limitations	Clinical Readiness and Key References
FC	S100A8/S100A9 from recruited intestinal neutrophils	Intestinal neutrophilic inflammation; correlation with endoscopic activity, generally stronger in UC	Within-patient, preanalytical, and assay variability; nonspecific elevation; lower/heterogeneous sensitivity in isolated or proximal small-bowel CD	Routine and guideline-supported; repeat or confirm unexpected values [2,3,7,8,15]
Fecal lactoferrin	Neutrophil secondary granules	Neutrophil-dominant intestinal inflammation	Less standardized and available; limited small-bowel evidence	Routine in selected settings [15,16,17]
CRP	Hepatic acute-phase response	Nonlocalizing systemic inflammation	May remain normal in active IBD, especially UC; nonspecific and host-dependent	Routine and guideline-supported [2,3,5,6]
ESR	Plasma proteins and erythrocyte aggregation	Slow systemic inflammatory signal	Affected by age, sex, anemia, immunoglobulins, and albumin	Routine adjunct; not a mucosal surrogate [6,18]
Albumin	Hepatic synthesis, distribution, catabolism, and protein loss	Subacute inflammatory or protein-loss burden	Long half-life; enteric/capillary loss; not a direct nutritional marker	Routine contextual marker [19]
Platelets	Megakaryopoiesis	Reactive thrombocytosis in systemic inflammation	Low specificity; iron deficiency is an independent driver	Routine contextual marker [20,21]
LRG	Hepatocytes, neutrophils, and inflamed tissue	Nonlocalizing multi-cytokine inflammation; may detect activity with normal CRP	Evidence largely Japanese; assays/cutoffs not internationally standardized; stenosis association is not fibrosis specificity	Emerging/regionally available; no biomarker-guided outcome evidence [14,22,23,24,25]
FIT	Human fecal globin	Gastrointestinal bleeding, mainly useful in UC/colonic disease	Nonspecific source; a negative result does not exclude nonbleeding inflammation	Context-specific adjunct, especially in UC [26,27,28]

Abbreviations: CD, Crohn’s disease; CRP, C-reactive protein; ESR, erythrocyte sedimentation rate; FC, fecal calprotectin; FIT, fecal immunochemical test; IBD, inflammatory bowel disease; LRG, leucine-rich alpha-2 glycoprotein; UC, ulcerative colitis. Clinical readiness refers to the role of each measure in current inflammatory bowel disease practice and does not imply universal assay availability.

**Table 2:** Recurrent discordance patterns and illustrative, context-dependent next assessments.

Pattern	Observed Mismatch	Probabilistic Interpretation	Illustrative Next Assessment (Context-Dependent)	Research-Only Adjuncts
A	High symptoms; low FC and CRP	Noninflammatory causes are more likely, but small-bowel, structural, infectious, or postoperative disease remains possible	Assessment of clinical context and functional or bile-acid causes; phenotype-directed endoscopy or imaging for persistent or red-flag symptoms; avoidance of reflex escalation [3,7,11]	None required
B	Low/absent symptoms; high FC	Subclinical neutrophilic activity is possible; infection, NSAIDs, and sampling variation are alternatives	Repeat testing when appropriate; exclusion of confounders; objective confirmation of persistent elevation by endoscopy or imaging before treatment change [2,3,43]	LRG or FIT may complement but not replace confirmation
C1	High CRP; low FC	Systemic, extraintestinal, infectious, penetrating/abscess, or small-bowel source is possible	Evaluation for non-IBD and extraintestinal sources; cross-sectional imaging when small-bowel or transmural disease is suspected [5,7]	LRG is nonlocalizing
C2	High FC; low CRP	Localized mucosal inflammation is possible despite a limited systemic response	Exclusion of stool-related confounders; endoscopy or phenotype-directed imaging before treatment change [2,5]	No validated localization marker
D	Normal endoscopy with active histology, or conventional remission with abnormal barrier function	Microscopic inflammation and functional barrier dysfunction are distinct	Biopsies for histology; CLE only in expert/research settings	Circulating barrier/repair markers are research only [44,45]
E	Low inflammatory markers with structural or transmural disease	Restricted or fibrostenosing/penetrating disease may be missed by routine markers	IUS/MRE or other phenotype-directed imaging; multidisciplinary review	Circulating ECM assays are research only [46,47,48]

Abbreviations: CLE, confocal laser endomicroscopy; CRP, C-reactive protein; ECM, extracellular matrix; FC, fecal calprotectin; FIT, fecal immunochemical test; IBD, inflammatory bowel disease; IUS, intestinal ultrasound; LRG, leucine-rich alpha-2 glycoprotein; MRE, magnetic resonance enterography; NSAID, nonsteroidal anti-inflammatory drug. Patterns A to E are unvalidated heuristic clinical combinations, not phenotypes, diagnostic rules, or treatment algorithms. Suggested assessments are illustrative and must be individualized according to pretest probability, disease phenotype, prior investigations, safety, and patient preference.

**Table 3:** Functional biomarker domains and complementary interpretive layers in inflammatory bowel disease: representative measures, test classes, predominant information, evidence maturity, and interpretation boundaries.

Category	Representative Measures	Test Class	Predominant Information	Evidence Maturity and Interpretation Boundary
Fecal–neutrophil biomarker domain	FC; fecal lactoferrin	Stool biomarkers	Intestinal neutrophil activity	Routine; location-dependent and nonspecific; no fibrosis or exposure information [2,3,30]
Serum–systemic biomarker domain	CRP; ESR; albumin; platelets; LRG	Serum biomarkers/contextual indices	Systemic inflammatory, protein, and hematologic response	Routine except LRG, which is emerging/region-specific; nonlocalizing and confounded [5,6,14]
Epithelial/barrier biomarker domain	I-FABP; citrulline; LBP; sCD14; LPS	Circulating investigational biomarkers	Enterocyte injury/mass; microbial-product response	Human-investigational; not permeability-equivalent; no treatment thresholds [52,53,54]
Restitution/resolution biomarker domain	REG1A/REG3A; IL-22; amphiregulin; TFFs; mucins; annexin A1; SPMs	Mechanistic/investigational measures	Regeneration and resolution pathway activity	No validated marker of completed repair; direction is context-dependent [55,56]
Fibrosis/ECM-remodeling biomarker domain	MMP-9; PRO-C3/PRO-C4/PRO-C6; C3M/C4M; YKL-40; galectin-3	Circulating investigational biomarkers	ECM turnover/remodeling, not fixed scar burden	Research only; insufficient fibrosis specificity and no diagnostic or treatment thresholds [46,47,57]
Symptom/functional overlay	Patient-reported symptoms; HBI; partial Mayo score	Clinical measures (not biomarkers)	Patient burden and noninflammatory overlap	Established outcomes; may diverge from objective inflammation [1,11]
Pharmacologic measurements	Drug concentrations; ADAs	Exposure/immunogenicity measurements	Exposure, clearance, and immunogenicity	Reactive anti-TNF TDM is most mature; non-anti-TNF thresholds remain unvalidated and assay/outcome-specific [58,59]
Reference and functional assessments	Endoscopy with biopsies; IUS; MRE; pelvic MRI; CLE/permeability tests	Reference/functional assessments	Mucosal, microscopic, transmural, structural, perianal, or barrier status	Adjudicates discordance; routine status is modality-specific; CLE/permeability testing is specialized [44,60,61,62]

Abbreviations: ADA, anti-drug antibody; C3M/C4M, matrix metalloproteinase-generated degradation markers of collagens III and IV; CLE, confocal laser endomicroscopy; CRP, C-reactive protein; ECM, extracellular matrix; ESR, erythrocyte sedimentation rate; FC, fecal calprotectin; HBI, Harvey–Bradshaw Index; I-FABP, intestinal fatty acid-binding protein; IL, interleukin; IUS, intestinal ultrasound; LBP, lipopolysaccharide-binding protein; LPS, lipopolysaccharide; LRG, leucine-rich alpha-2 glycoprotein; MMP, matrix metalloproteinase; MRE, magnetic resonance enterography; PRO-C3/PRO-C4/PRO-C6, formation markers of collagens III, IV, and VI; MRI, magnetic resonance imaging; REG, regenerating islet-derived protein; sCD14, soluble CD14; SPM, specialized pro-resolving mediator; TDM, therapeutic drug monitoring; TFF, trefoil factor; TNF, tumor necrosis factor; YKL-40, chitinase-3-like protein 1. Evidence-maturity categories are descriptive rather than formal certainty ratings. The five biomarker domains are functional interpretive categories based on predominant readout; they overlap biologically and do not represent mutually exclusive compartments.

## Data Availability

No new data were created or analyzed in this study. Data sharing is not applicable to this article.

## Abbreviations

The following abbreviations are used in this manuscript:

ADA	Anti-drug antibody
anti-TNF	Anti-tumor necrosis factor
AUC	Area under the curve
C3M/C4M	Matrix metalloproteinase-generated degradation markers of collagens III and IV
CD	Crohn’s disease
CLE	Confocal laser endomicroscopy
CRP	C-reactive protein
ECM	Extracellular matrix
ESR	Erythrocyte sedimentation rate
FC	Fecal calprotectin
FIT	Fecal immunochemical test
IBD	Inflammatory bowel disease
I-FABP	Intestinal fatty acid-binding protein
IL-22	Interleukin-22
IUS	Intestinal ultrasound
LBP	Lipopolysaccharide-binding protein
LPS	Lipopolysaccharide
LRG	Leucine-rich alpha-2 glycoprotein
MMP-9	Matrix metalloproteinase-9
MRE	Magnetic resonance enterography
MRI	Magnetic resonance imaging
NSAID	Nonsteroidal anti-inflammatory drug
PRO-C3/PRO-C4/PRO-C6	Formation markers of collagens III, IV, and VI
REG1A/REG3A	Regenerating islet-derived proteins 1 alpha and 3 alpha
sCD14	Soluble CD14
SPMs	Specialized pro-resolving mediators
TDM	Therapeutic drug monitoring
TFFs	Trefoil factors
UC	Ulcerative colitis
YKL-40	Chitinase-3-like protein 1
